# Path planning of a manipulator based on an improved P_RRT* algorithm

**DOI:** 10.1007/s40747-021-00628-y

**Published:** 2022-01-21

**Authors:** Junhui Yi, Qingni Yuan, Ruitong Sun, Huan Bai

**Affiliations:** Key Laboratory of Modern Manufacturing Technology, Ministry of Education, West Campus, Guizhou University, Huaxi District, Guiyang, Guizhou China

**Keywords:** Manipulator, Path planning, Improved P_RRT* algorithm, Nearest neighbour node selection, Two expansions

## Abstract

Aiming to build upon the slow convergence speed and low search efficiency of the potential function-based rapidly exploring random tree star (RRT*) algorithm (P_RRT*), this paper proposes a path planning method for manipulators with an improved P_RRT* algorithm (defined as improved P_RRT*), which is used to solve the path planning problem for manipulators in three-dimensional space. This method first adopts a random sampling method based on a potential function. Second, based on a probability value, the nearest neighbour node is selected by the nearest Euclidean distance to the random sampling point and the minimum cost function, and in the expansion of new nodes, twice expansion methods are used to accelerate the search efficiency of the algorithm. The first expansion adopts the goal-biased expansion strategy, and the second expansion adopts the strategy of random sampling in a rectangular area. Then, the parent node of the new node is reselected, and the path is rerouted to obtain a clear path from the initial point to the target point. Redundant node deletion and the maximum curvature constraint are used to remove redundant nodes and minimize the curvature on the generated path to reduce the tortuosity of the path. The Bezier curve is used to fit the processed path and obtain the trajectory planning curve for the manipulator. Finally, the improved P_RRT* algorithm is verified experimentally in Python and the Robot Operating System (ROS) and compared with other algorithms. The experimental results verify the effectiveness and superiority of the improved algorithm.

## Introduction

**Fig. 1:**
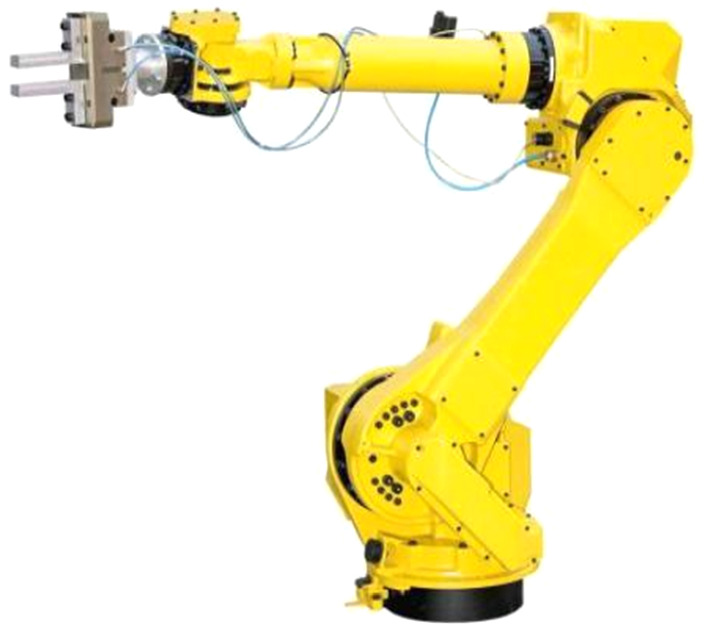
Storage robotic arm

**Fig. 2:**
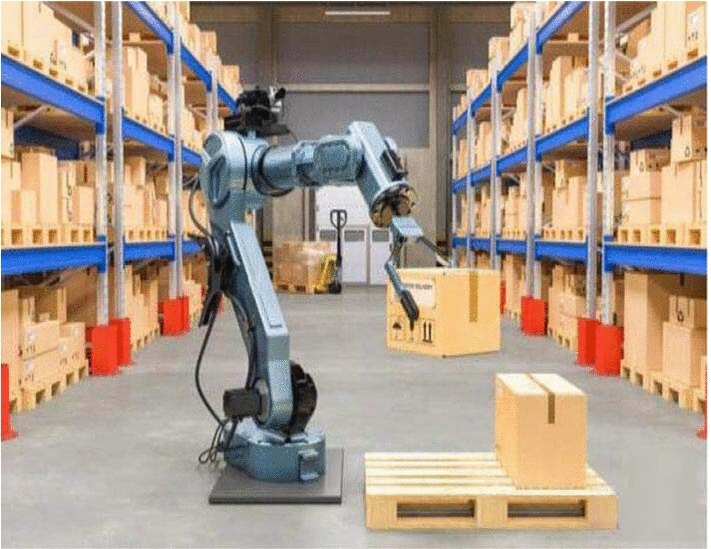
Robotic arm is used to carry goods

With the introduction of German Industry 4.0, China has successively proposed the “Made in China 2025” strategy to adapt to the gradually increasing requirements for the intelligentization of robotic arms. The term robotic arm is short for robotic intelligent arms. As the name implies, a robotic arm is an important bionic arm that imitates human arms, as shown in Fig. 1. The roles of robotic arms are important and include lifting, transporting, grasping, and loading heavy objects; they are also used in high-risk, high-precision processing operations and other fields [1], as shown in Fig. 2. At present, China’s industrial robotic arms are mainly used in factories. The logistics industry has developed rapidly in recent years, requiring considerable manpower and material resources, and intelligent warehousing technology has developed rapidly. Robotic arms play a vital role in the intelligent warehousing industry and can be used to reduce production costs and resource waste; they have always been a hot spot in robotics research [2, 3]. Among existing studies in robotic arm research, the motion stability of a robotic arm is a key topic, so developing a method to find an efficient and collision-free path is particularly important for the motion of a robotic arm [4].

Current motion planning algorithms are based on the grid search method (A*), artificial potential field method (APF), probabilistic roadmap method (PRM) and rapidly exploring random tree (RRT) algorithm [5]. Among them, the grid search method can ensure complete resolution and an optimal solution in path planning, but the flexibility of the algorithm is limited and the calculation efficiency is low; the artificial potential field method is prone to local minima in the path planning algorithm, resulting in the inability to reach the target point; the probability map method of the multiquery method is probabilistically complete in path planning, but it requires the state space to be known in advance and has low efficiency; the RRT algorithm poses problems, such as large memory usage and low search efficiency in a complex multiobstacle environment [6, 7]. The RRT algorithm randomly samples in the state space without preprocessing the state space. Its randomness proves that the RRT algorithm itself has strong search capabilities and that the algorithm is probabilistically complete. Many scholars have conducted in-depth research on the RRT algorithm [8, 9].

In view of the shortcomings of the RRT algorithm, a variety of RRT algorithm variants and other algorithms have emerged to improve the efficiency of path planning. Pérez-Higueras et al. presented an approach for learning navigation behaviours for robots using an optimal rapidly exploring random tree star (RRT*) as the main planner. A new learning algorithm combining both inverse reinforcement learning and RRT* is developed to learn RRT*’s cost function from demonstrations [10]. Li et al. proposed an incremental sampling-based motion planning algorithm, i.e., near-optimal RRT (NoD-RRT). This algorithm aims to solve motion planning problems with nonlinear kinodynamic constraints. To determine the cost/metric between two given states considering the nonlinear constraints, a neural network is utilized to predict the cost function. On this basis, a new reconstruction method for the random search tree is designed to achieve a near-optimal solution in the configuration space [11]. Chen et al. proposed a novel approach of RRT* in collaboration with a double-tree structure to separate the extension and optimization procedures [12]. Hidalgo-Paniagua et al. presented quad-RRT, an extension of the bidirectional strategy to speed up the RRT when dealing with large-scale, bidimensional (2D) maps [13]. Chao et al. proposed an algorithm called grid-based RRT* (GB-RRT*) by combining the principle of RRT* with the grid searching strategy. The proposed hybridized algorithm compensates for the weaknesses of RRT* with the strengths of the grid search strategy and is applicable in complex environments with obstacles and narrow areas without relying on predesigned road networks [14]. Ryu et al. proposed an improved informed RRT* algorithm to reduce computational time, even in complex environments. Unlike the use of RRT* for informed RRT* to find an initial solution for the whole configuration space, the gridmap skeletonization approach is applied to generate the initial solution [15]. Kiani et al. presented an improvement on the RRT algorithm, namely, Adapted-RRT, which uses three well-known metaheuristic algorithms, namely, grey wolf optimization (GWO), incremental grey wolf optimization (IGWO), and expanded grey wolf optimization (Ex-GWO). They attempted to find solutions close to the optima without collision while providing comparatively efficient execution time and space complexities [16]. Qi et al. presented an algorithm termed multiobjective dynamic rapidly exploring random (MODRRT*), which is suitable for robot navigation in an unknown dynamic environment [17]. Qureshi et al. added the idea of the artificial potential field method to the RRT* algorithm to obtain the P-RRT* algorithm, which significantly improves the shortcoming of slow convergence efficiency of the algorithm and minimizes the use of memory [18]. Jeong et al. proposed the Q-RRT* algorithm, which also considers the parent node of a new node’s parent node on the basis of RRT*, expands the ranges of parent vertices, and uses this method in the rewiring operation. This method further improves the search efficiency of the algorithm and the quality of the path [9]. Li et al. proposed a PQ-RRT* algorithm that combines the potential function-based RRT* (P_RRT*) and quick RRT* (Q_RRT*) algorithms to fully utilize the advantages of the two algorithms and further improve the convergence rate and efficiency of the algorithm [7]. Hu et al. proposed a learning scheme with nonlinear model predictive control (NMPC) for mobile robot path tracking. The NMPC strategy utilizes a varying-parameter one-layer projection neural network to solve an online quadratic programming optimization via iteration over a limited receding horizon [19]. Hu et al. developed a 9-degree-of-freedom (DOF) rigid-flexible coupling (RFC) robot to assist coronavirus disease 2019 (COVID-19) oropharyngeal (OP) swab sampling. Compared with a rigid sampling robot, the developed force sensing RFC robot can facilitate OP swab sampling procedures in a safer and more gentle way. In addition, a varying-parameter zeroing neural network-based optimization method was proposed for motion planning of the 9-DOF redundant manipulator [20]. Jordan et al. proposed bidirectional RRT* (B-RRT*) to use the greedy connection heuristic to connect two directional trees to improve the efficiency of the algorithm. However, the algorithm only explores the pure space, and the expansion of the random tree is blind [21]. Wei et al. proposed smooth RRT (S-RRT) and adopted a path optimization strategy based on the maximum curvature constraint to generate a smooth and curved continuous executable path for the robot manipulator. However, for the expansion of the random tree, only the idea of target bias is used for expansion, the search efficiency is still low, and there is a large difference between the fitted path and the original path [22]. Nasir et al. proposed RRT*-Smart, and two new technologies, path optimization and intelligent sampling, were used to improve the efficiency of the RRT*-Smart algorithm. The algorithm only experiments in a two-dimensional environment, the generated path is relatively tortuous, and the proposed method cannot improve the efficiency of the algorithm very well [23]. As combinations of the above algorithms, these algorithms have improved the efficiency and convergence of the path search to a certain extent. In the face of complex obstacle environments, the above algorithms still have low efficiency, slow convergence to the optimal solution, and large computational memory usage. For the motion path of a robotic arm, there are many turning points, and the planned path is not smooth, which seriously affects the service life of the robotic arm.

In response to the above problems, this paper proposes an improved P_RRT* algorithm for path planning of manipulators. This method is used to achieve efficient path planning for manipulators in a complex obstacle environment. Compared with the existing path planning algorithm, the main contributions of the text are as follows:

1. For the selection of the nearest neighbour nodes, two methods, the minimum Euclidean distance and the minimum cost function, are proposed to improve the convergence speed of the algorithm.
2. For the expansion of new nodes, a two-step expansion strategy is proposed. The first step expansion adopts the idea of target bias for expansion, and the second step expansion adopts the method of random sampling of rectangular regions. This expansion method is used to improve the search speed of the algorithm.
3. Since the generated path has many inflection points and the path curvature is large, the removal operation for redundant nodes and the constraint operation of maximum curvature are used to make the path smoother.

The rest of this article is arranged as follows: The “Definition of the path planning problem” section introduces the basic definition and the problems that need to be solved for path planning; “Basic P_RRT* algorithm” introduces the background knowledge of the improved algorithm presented in this article; “Improved P_RRT* algorithm” introduces the specific implementation steps of the improved algorithm presented in this article; “Analysis” introduces the analysis and optimal solution of the path planning problem; “Deletion of redundant nodes and the maximum curvature constraint” introduces the smoothing operation of the generated path, which includes the deletion of redundant nodes in the path and the maximum curvature constraint operation on the path; “Bezier curve” introduces the use of the Bezier curve to fit the final path; “Experiments and analysis” introduces the Python and robot operating system (ROS) simulation experiment analyses of the improved algorithm presented in this article; “Conclusion” summarizes this article.

## Definition of the path planning problem

This section introduces three problems that need to be solved in path planning. Let *Q* $\subset$ *R*^n^ denote the state space of the problem described, where *R* is a set, and *r*(*i*)_*i*∈*n*_ denotes the mapping from *n* to *R*; that is, *i* $\in$ *n* is mapped to *r*(*i*) $\in$ *R*. *Q*_obs_ $\subset$ *Q* denotes the obstacle space, and *Q*_free_ = *Q*\*Q*_obs_ denotes the barrier-free space. *Q*_init_ and *Q*_end_ are the starting state and the ending state, respectively. They are both in an obstacle-free space. The obstacle spatial distance (d^*^_obs_) is set: that is, the obstacle model is simplified to a sphere, where the distance that the radius of the sphere expands outward is used. The continuous function *α*: [0,1] $\to$ *Q*_free_ is a feasible collision-free path [24].

The problem of path planning is to find a collision-free path, namely, *α*: [0,1]$\to$*Q*_free_. The path that extends from the starting point *α* (0) $\in$ *Q*_init_ to the ending point *α* (1) $\in$* Q*_end_ and through the point *α*(*τ*) $\in$ *Q*_free_ for all *τ* $\in$ [0,1] is called a feasible path.

Question 1: For path planning problem {*Q*_init_, *Q*_obs_, *Q*_end_}, if a path is found, it proves that there is a feasible path, and if the path is not found, it proves that a path does not exist.

Question 2: For all the path sets ∑, ∑_feasible_ represents all feasible paths, and *C*(.) represents the cost function measured by the Euclidean distance; then, given the path planning problem {*Q*_init_, *Q*_obs_, *Q*_end_}, find a path α* $\in$ ∑_feasible_; if there is *C* (α*) = min{*C* (*α*): *α* $\in$ ∑_feasible_}, there is an optimal path; otherwise, the optimal path planning process fails.

Question 3: For *t* $\in$ *T*, which represents the time required for the algorithm to find a collision-free path, the algorithm is required to find the optimal path in the shortest time *t* $\in$ *T* [7, 9, 18].

## Basic P_RRT* algorithm

**Fig. 3:**
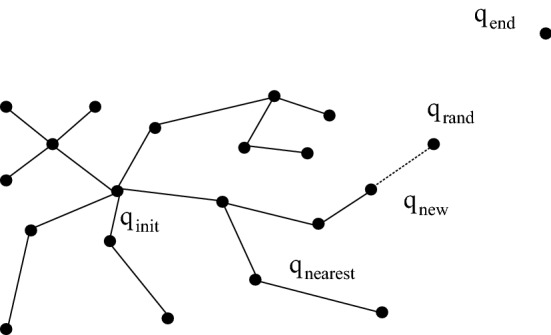
Basic RRT algorithm mind map

The classic RRT algorithm [5] is an efficient multidimensional space planning method. An initial node is taken as the root node (*q*_init_); then, a random sampling point (*q*_rand_) in the state space.

is selected, and the node (*q*_nearest_) in the random tree that is closest to *q*_rand_ in terms of the Euclidean distance according to the Get_Nearest function is selected. Then, *q*_nearest_ is extended to *q*_rand_ by a step (*ρ*) distance, a new node *q*_new_ is generated, and it is judged whether *q*_new_ collides with any obstacles. If there is a collision, *q*_new_ is deleted, and the random points are resampled. If there is no collision, *q*_new_ is added to the random tree, and the parent node of *q*_new_ is assigned as *q*_nearest_, as shown in Fig. 3. The next random sampling is performed, and the above steps are repeated. When the distance between *q*_new_ and the target point *q*_end_ is less than the step length *ρ*, *q*_new_ and the target point are connected to obtain a collision-free path from the starting point to the ending point.

**Fig. 4:**
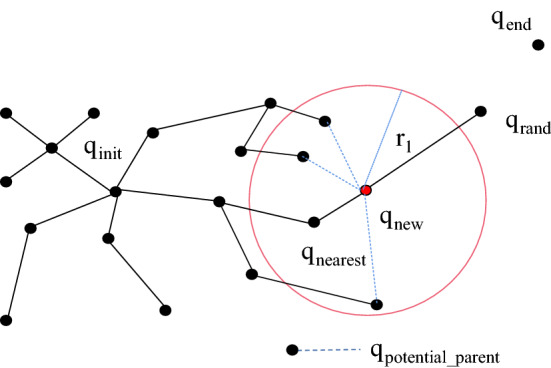
RRT* algorithm is used to reselect the mind map of the parent node

**Fig. 5:**
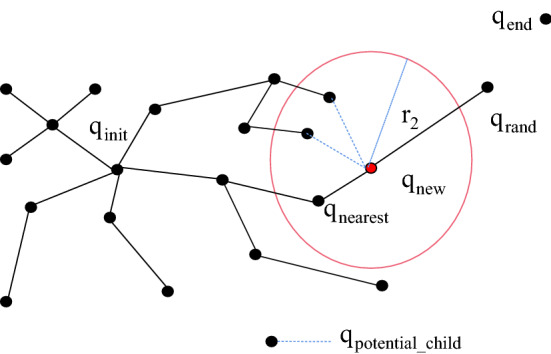
RRT* algorithm for the rewiring mind map operation

The RRT* algorithm [25] is an improved algorithm based on the traditional RRT algorithm; it reselects the parent node in the nearest neighbour area (Reselect_Parent_Node) and rewires the node in the nearest neighbour area (Rewire_Node). In the RRT algorithm, the parent node of *q*_new_ is assigned as *q*_nearest_, and the RRT* algorithm improves the selection of the parent node of the new node. Specifically, the set (*Q*(potential_parent)) of all the adjacent potential parent nodes of q_new_ in the tree within a circle, where *q*_new_ is the centre and *r*_1_ is the radius, is found. Then, whether there is a node in *Q*(potential_parent) that is more suitable than *q*_nearest_ to be the parent node of *q*_new_ is determined; that is, the Euclidean distance from *q*_new_ to *q*_init_ is determined and used as the cost function. This method first calculates the Euclidean distance cost value, Cost(*q*_new_), with *q*_nearest_ as the parent node, selects the potential parent node in *Q*(potential_parent) to connect with the new node, and then judges the potential parent node as the parent of the new node. It is determined whether the Euclidean distance cost value of the node, Cost(*Q*(potential_parent)), is less than Cost(*q*_new_); if it is greater than Cost(*q*_new_), the potential parent node will be discarded, and the next potential parent node will be calculated. If it is less than Cost(*q*_new_) and the connection between the potential parent node and the new node does not collide with an obstacle, then the parent node of *q*_new_ is reselected and connected. If it collides with an obstacle, the parent node of the new node remains unchanged as *q*_nearest_. The nodes are compared in sequence until all potential parent nodes are compared, as shown in Fig. 4. The RRT* algorithm also uses the rewiring of nodes in the nearest neighbour area. The idea is to use *q*_new_ as the parent node of all potential child nodes Q(potential_child) in an adjacent circle with *r*_2_ as the radius. If the Euclidean distance cost value, Cost(*Q*(potential_child)), of a parent node with *q*_new_ as a potential child node is smaller than the original Euclidean distance cost value of the potential child node and if it does not collide with obstacles, the original parent node of the potential child node is discarded, and the new node is used as its parent node for rewiring; otherwise, the parent node remains unchanged, as shown in Fig. 5. The codes for reselecting the parent node and the rewiring operation of the RRT* algorithm are shown in Algorithm 1 and Algorithm 2, respectively. The Euclidean distance cost value is the sum of the Euclidean distance of the path from this point to the root node.

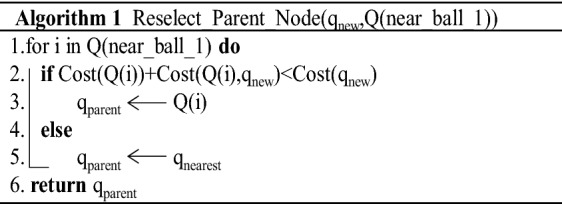

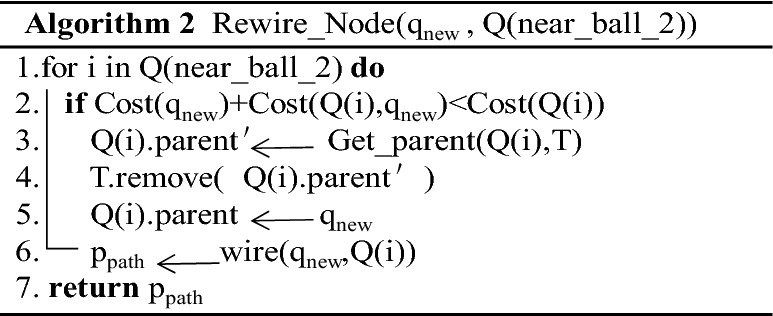

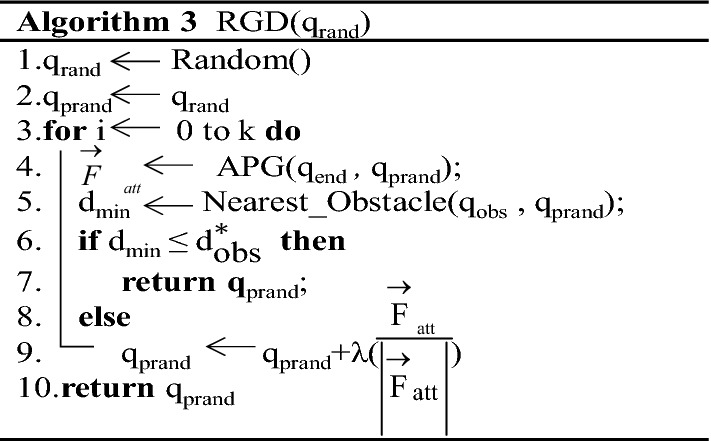







The P_RRT* algorithm uses a potential function to improve the selection of random sampling points *q*_rand_ on the basis of RRT* and is defined as the potential function random sampling point selection method (RGD(*q*_rand_)), and the RGD(*q*_rand_) code is shown in Algorithm 3. Specifically, RGD(*q*_rand_) introduces the idea of the artificial potential field method in the operation of selecting random nodes (*q*_rand_). APG stands for the attraction gradient [18]. The target point generates attraction, and a potential random sample point is defined as *q*_prand_. *q*_prand_ is a point from *q*_rand_ along the decreasing direction of the attractive potential field gradient; that is, the point is biased towards the target direction, and it is obtained by moving downhill with a small step *λ*. When *q*_prand_ is used instead of *q*_rand_ as a random sampling point, the potential field gradient of gravity will decrease upon approaching the target point. During the expansion process, the Euclidean distance to the nearest obstacle is judged in real time. If it is less than the obstacle spatial distance (d*_obs_), the program will be terminated immediately, and *q*_prand_ will be returned. Otherwise, the next expansion will be performed with a limited number of k, in which the values of the parameters *λ*, *k* and d*_obs_ need to be adjusted. This article ignores the adjustment of the parameters and sets *λ* = 0.02, *k* = 80, and d*_obs_ = 0.1.

## Improved P_RRT* algorithm

The basic P_RRT* algorithm improves the selection of random nodes in the state space, which makes it more biased towards the location of the target point, reduces the probability of generating invalid nodes in a tree, saves calculation space and reduces memory usage. However, the efficiency of a successful path search and the convergence rate of a search are still very low. To improve the search efficiency and achieve a higher convergence rate, in this paper, the selection of nearest neighbour nodes and the expansion of new nodes in the P_RRT* algorithm are further improved.

### Selection of the nearest neighbour node

This paper improves the selection of the nearest neighbour node in the P_RRT* algorithm (Nearest_Neighbour(*q*_prand_,*q*_end_)). The traditional nearest neighbour node (*q*_nearest_) selection method selects the node with the closest Euclidean distance to the random sampling point as the nearest neighbour node (*q*_nearest_). In this paper, searching for the smallest value of the cost function *C*(*q*) in the tree as the nearest neighbour node (*q*_nearest_), *C*(*q*) is defined as

1 $$ C\left( q \right) = w_{d}  \times q_{{{\text{end}}}}  - q+ w_{c}  \times \left( {\frac{k}{{\Vert {q_{{{\text{end}}}}  - q}\Vert }}} \right) $$

where $\Vert {q}_{\text{end}}\text{ } - \Vert {q}$ is the Euclidean distance between the target point *q*_end_ and the current node *q*, *w*_d_ is the distance proportional coefficient, *w*_c_ is the mixed proportional coefficient, and k represents the number of obstacles in the circle near the current node. The values of *w*_d_ and *w*_c_ are obtained through the distribution of obstacles and experimental analysis. In this article, the values are *w*_d_ = 1 and *w*_c_ = 4. To improve the ability of the model to avoid obstacles, the radius of the circle that determines the value of *k* in formula (1) is twice the length of r_1_ in the RRT* algorithm, and the nearest neighbour node chooses the smallest *C* (*q*) in the tree.
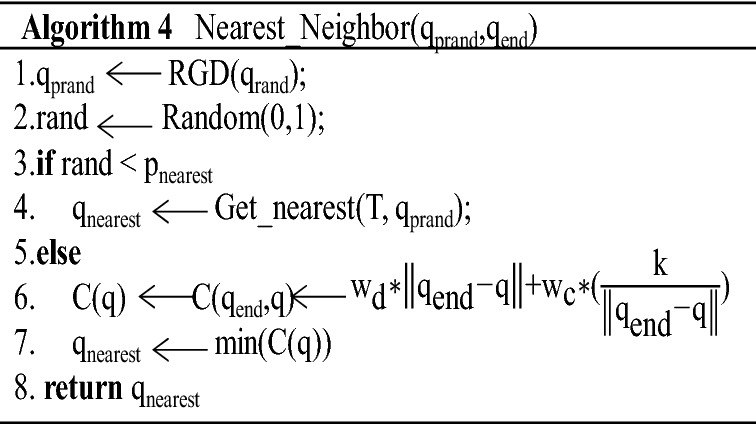


Let rand be a uniformly distributed random number in the interval [0,1]. To improve the obstacle avoidance ability of the algorithm, according to the experimental analysis, the value of *p*_nearest_ = 0.5. *P*_nearest_ represents the probability of selecting the node with the smallest Euclidean distance from the random sampling point in the tree as the *q*_nearest_; that is, if rand < *p*_nearest_, then the node with the smallest Euclidean distance from the random sampling point (*q*_prand_) is selected as the nearest neighbour node (*q*_nearest_). If rand > *p*_nearest_, the point with the smallest cost function *C* (*q*) in the tree is selected as the nearest neighbour node (*q*_nearest_). The corresponding code is shown in Algorithm 4.

### Expansion of new node

**Fig. 6:**
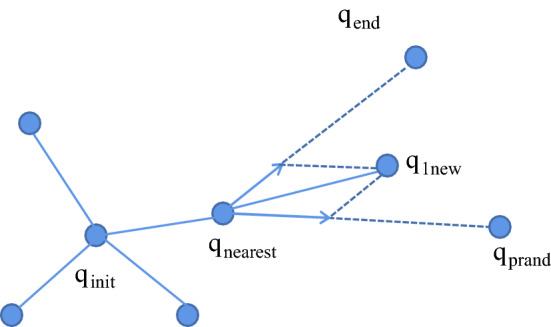
q1new expansion mind map


The traditional P_RRT* algorithm first samples random points based on the potential function, finds the node (*q*_nearest_) closest to the random node and expands along the direction of the random point to obtain *q*_new_. The traditional algorithm only expands the new node once, which is not efficient. This article improves the expansion of the new node in the P_RRT* algorithm, and the strategy of expanding the new node twice is adopted to speed up convergence and improve the efficiency of the algorithm. The specific steps are as follows:

The new node (*q*_1new_) in the first step is expanded with the goal-biased expansion method [26, 27]: that is, the idea of gravitation in the artificial potential field method is introduced on the expanded step size so that it grows towards the target point, as shown in Fig. 6. *q*_1new_ is defined as:

2 $$ q_{{{\text{1new}}}} = q_{{1{\text{new}}}} + \rho *\left( {\frac{{q_{{{\text{prand}}}} - q_{{{\text{nearest}}}} }}{{q_{{{\text{prand}}}} - q_{{{\text{nearest}}}} }} + k_{p} *\frac{{q_{{{\text{end}}}} - q_{{{\text{nearest}}}} }}{{q_{{{\text{end}}}} - q_{{{\text{nearest}}}} }}} \right). $$

where *k*_p_ is the gravitational coefficient, *ρ* is the growth step of the random tree, *q*_prand_ is the random sampling point, *q*_nearest_ is the nearest neighbour node, $\Vert {q}_{\text{end}}-{q}_{\text{nearest}}\Vert$ is the Euclidean distance from the target point (*q*_end_) to the nearest neighbour node (*q*_nearest_), and $\Vert {q}_{\text{prand}}-{q}_{\text{nearest}}\Vert$ is the Euclidean distance from the random sampling point (*q*_prand_) to the nearest neighbour node (*q*_nearest_). Whether the path between *q*_1new_ and *q*_nearest_ collides with obstacles is determined; if there is a collision, the expansion of *q*_1new_ will be abandoned, and the random sampling point will be selected again using the method of selecting a random sampling point in the P_RRT* algorithm. If there is no conflict, *q*_1new_ is added to the random tree, and its parent is assigned to *q*_nearest_.

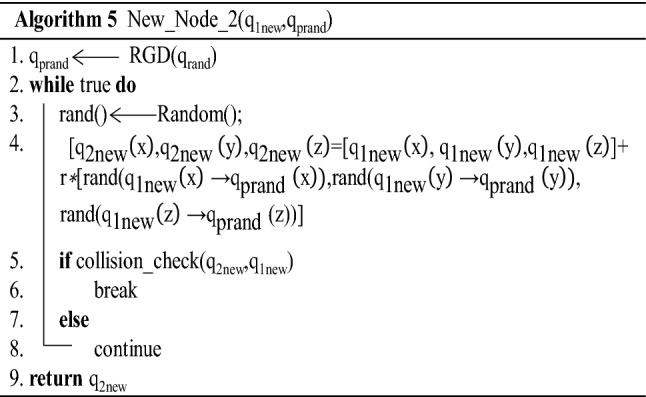


**Fig. 7:**
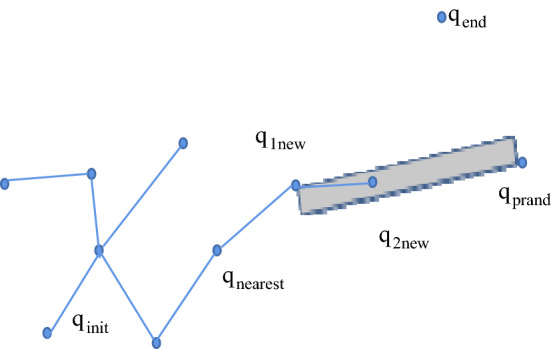
q2new expansion mind map

The second step of the expansion of the new node (*q*_2new_) adopts the strategy of random sampling from *q*_1new_ to *q*_prand_ in a rectangular area (New_Node_2(*q*_1new_,*q*_prand_)). That is, a point is randomly selected in the rectangular area formed by the new node q_1new_ of the first expansion to the last expansion point *q*_rand_ and is assigned as the new node *q*_2new_ of the second expansion, as shown in Fig. 7. Let rand(*x*) be a randomly selected value in the range of *x*; then, the new node (*q*_2new_) can be expressed as:

3 $$ \begin{aligned}   {}& [q_{{{\text{2new}}}} \left( x \right),q_{{{\text{2new}}}} \left( y \right),q_{{{\text{2new}}}} \left( z \right)] \\   & \quad  = [q_{{{\text{1new}}}} \left( x \right),q_{{{\text{1new}}}} \left( y \right),q_{{{\text{1new}}}} \left( z \right)] + r{\text{*}}[{\text{rand}} \\   & \qquad\quad (q_{{{\text{1new}}}} \left( x \right) \to q_{{{\text{prand}}}} \left( x \right)){\text{,rand(}}q_{{{\text{1new}}}} \left( y \right) \to q_{{{\text{prand}}}} \left( y \right)), \\   & \qquad\quad {\text{rand(}}q_{{{\text{1new}}}} \left( z \right) \to q_{{{\text{prand}}}} (z))]\end{aligned}   $$

In the formula, *q*_2new_(), *q*_1new_(), and *q*_prand_() represent the coordinate values of *q*_2new_, *q*_1new_, and *q*_prand_, respectively; *r* represents the expansion ratio coefficient; and *r* = 0.01 according to the experimental analysis. In addition, whether the connection between *q*_2new_ and *q*_1new_ collides with obstacles is determined; if they collide, the expansion of *q*_2new_ is abandoned, and *q*_2new_ is resampled. If there is no collision, *q*_2new_ is added to the random tree, and its parent node is assigned to *q*_1new_. The code is shown in Algorithm 5.

### Implementation of the improved algorithm

According to the principle of the above improved P_RRT* algorithm, the specific implementation steps can be summarized as follows:

- Step 1: Initialize each parameter in the random tree, including inserting the starting point, target point, obstacles, step length, target bias step length, and obstacles;
- Step 2: Use the random sampling point selection method RGD (*q*_rand_) of the potential function in the P_RRT* algorithm to obtain the randomly sampled point *q*_prand_;
- Step 3: Obtain the random number probability (rand) that obeys the uniform distribution, and select the nearest neighbour node (*q*_nearest_). If the random number probability (rand) is less than the nearest neighbour node probability *p*_nearest_ = 0.5, then the nearest neighbour node is the node in the tree that has the closest Euclidean distance to the random sampling point (*q*_prand_); if the random probability rand is greater than *p*_nearest_ = 0.5, formula (1) is used to calculate the cost function *C* (*q*), and the point with the smallest cost function *C* (*q*) in the tree is selected as the nearest neighbour node;
- Step 4: Use the obtained nearest neighbour node for expansion. The first step is to expand and use the target bias strategy, obtain q_1new_ through formula (2), and judge whether the new node q_1new_ collides with obstacles. If collision with an obstacle occurs, then return to Step 2; otherwise, go to Step 5;
- Step 5: On the expanded new node q_1new_, the second step of expansion is carried out by formula (3), which uses a randomly selected point in the rectangular area as the second expanded new node to obtain *q*_2new_ and judges whether the new node *q*_2new_ collides with an obstacle or whether the line between two new nodes (*q*_1new_, *q*_2new_) collides with an obstacle. If there is a collision, execute Step 5 to randomly sample the new node q_2new_ again; otherwise, execute Step 6;
- Step 6: Perform the operation of reselecting the parent node in the RRT* algorithm for the new node in the adjacent circle (see Algorithm 1) and find the parent node of the new node with the smallest Euclidean distance cost value that does not collide with an obstacle;
- Step 7: The new node acts as the parent node of the node in the adjacent circle to perform the rewiring operation in the adjacent circle in the RRT* algorithm (see Algorithm 2). Find the node in the adjacent circle whose Euclidean distance cost value with the new node as its parent node is less than its own Euclidean distance cost value and that does not collide with an obstacle to perform the rerouting operation to find the optimal path;
- Step 8: Check whether the Euclidean distance between the new node and the target point is less than the step length *ρ*. If it is, connect the new node and the target point to obtain a complete and clear path, and the algorithm ends; otherwise, go to Step 2.

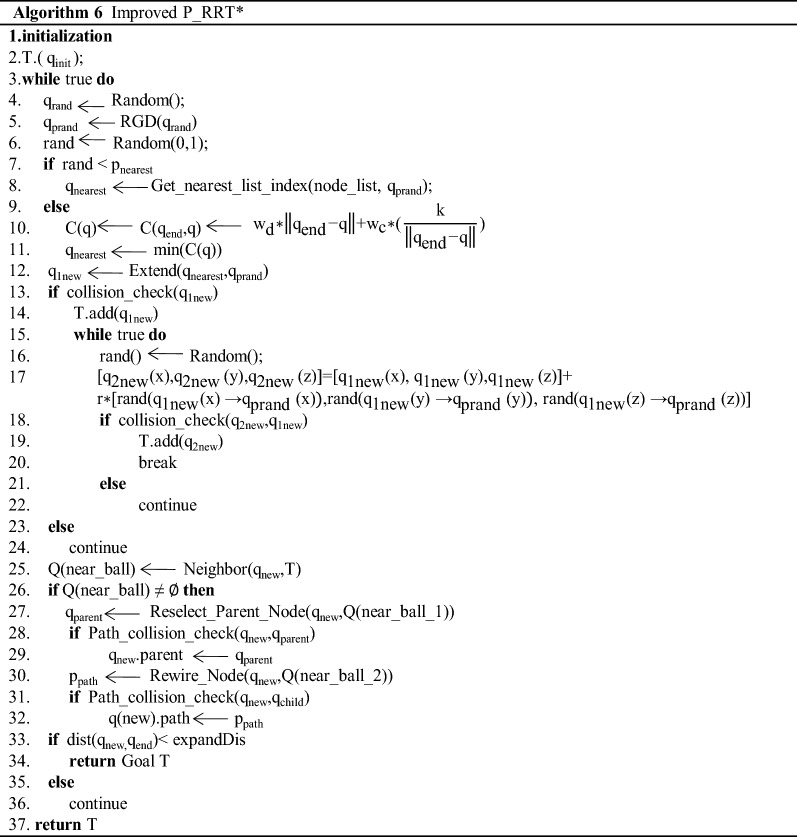


**Fig. 8:**
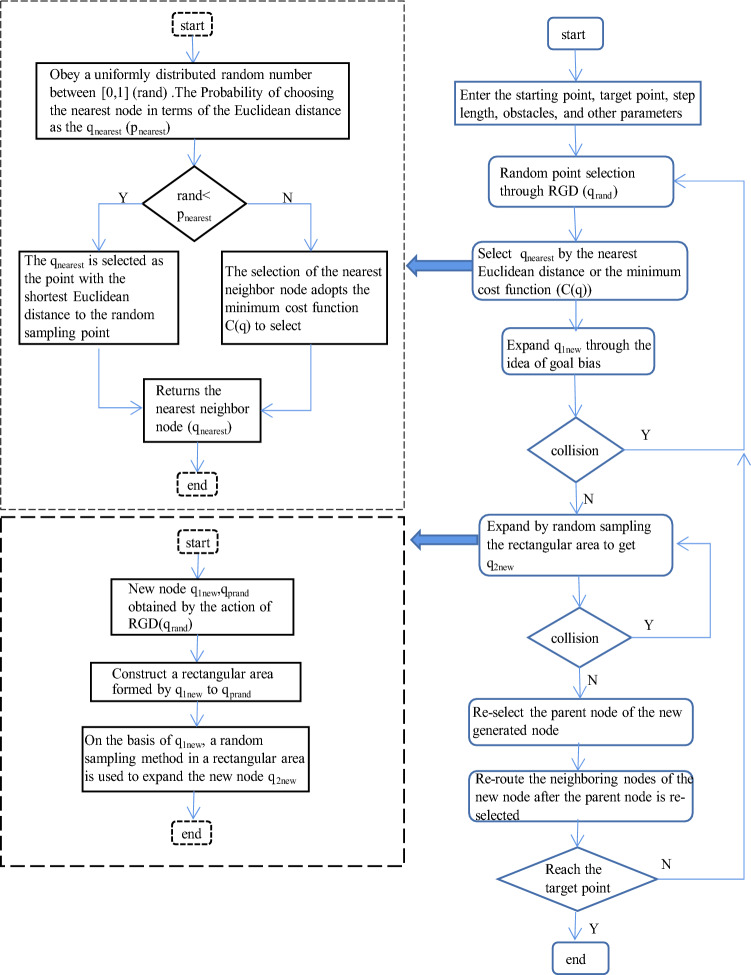
Improved P_RRT* algorithm flow chart

According to the above steps, the flow chart of the improved P_RRT* algorithm is shown in Fig. 8, and the code of the algorithm is shown in Algorithm 6.

### Analysis

This section analyses the probabilistic completeness, asymptotic optimality, fast convergence, and computational complexity of the improved P_RRT* algorithm. Let ALG represent an algorithm. ${{V}_{n}}^{\text{ ALG}}$ represents the vertex of the tree generated after *n* iterations of the ALG algorithm. ${Y}_{n}^{\text{ ALG}}$ represents the minimum path cost after n iterations of the ALG algorithm. ${{S}_{n}}^{\text{ ALG}}$ represents the number of steps after *n* iterations of the ALG algorithm.

### Probabilistic completeness

For Question 1 (mentioned in the definition of the path planning problem), the probabilistic completeness of most sampling-based algorithms can be guaranteed. The form of probabilistic completeness is as follows:

#### Definition 1

(*Probabilistic completeness*) For path planning problem {*Q*_init_, *Q*_obs_, *Q*_end_}, if the probability of finding a feasible path as the number of iterations increases is 1 for ALG, that is,

$$  \mathop {\lim }\nolimits_{{n \to 8}} \mathbb{P}(V_{n}^{{{\text{ALG}}}} \cap Q_{{{\text{end}}}}  \ne {\emptyset} ) = 1   $$

then the ALG algorithm generates a complete path connecting root *q*_init_ to *q*_end_ $\in$ *Q*_end_. The ALG algorithm has the characteristics of probabilistic completeness.

The probabilistic completeness of the RRT algorithm has been proven in detail, and the RRT* algorithm, as a variant of RRT, inherits the probabilistic completeness of the RRT algorithm [25]. At the same time, the probabilistic completeness of the P_RRT* algorithm has also been explained [18]. This paper explains the probabilistic completeness of the improved P_RRT* algorithm proposed in Theorem 1.

#### Theorem 1

(Probabilistic completeness of improved P_RRT*) For any feasible path planning problem {*Q*_init_, *Q*_obs_, *Q*_end_}, as the number of iterations increases, the probability of finding a feasible path is close to 1, that is,

$$ \begin{aligned} & \lim \nolimits_{{n \to 8}} {\mathbb{P}}(\exists q_{{{\text{end}}}}  \in V_{n}^{{{\text{ImprovedP\_RRT*}}}} \cap Q_{{{\text{end}}}} {\text{ such that }}q_{{{\text{init}}}} \\ &\quad{\text{ is connected to }}q_{{{\text{end}}}}  \in Q_{{{\text{end}}}} ) = 1   \end{aligned} $$

#### Proof of Theorem 1

This article uses the same sampling method as the P-RRT* algorithm, and the improved P_RRT* algorithm only improved the selection of its nearest neighbour nodes and the expansion strategy on the basis of the P_RRT* algorithm, which changed the growth trend of the tree but did not change the connectivity of the tree. Therefore, the improved P_RRT* algorithm has the same probabilistic completeness as the P_RRT* algorithm.

### Asymptotic optimality

For Question 2 (mentioned in the definition of the path planning problem), if the algorithm has a continuous path minimum cost solution α*: [0,1] such that α* (0) = *Q*_init_ and α*(1) $\in$ *Q*_end_, and there is no collision in the complex obstacle environment, then the algorithm is asymptotically optimal.

Let *β* $\in$ *R*_+_, for the state *q* $\in$ *Q*_free_, where *H*_q, β_ represents the closed ball area with radius β centred on q. If the ball area is completely located in the barrier-free space, then for any *q* $\in$ *Q*_free_, it can be expressed as β-internal state (*Q*_intβ_); and if the ball area is partially located in the barrier-free space, for any *q* $\in$ *Q*_free_, it can be defined as β-external state (*Q*_extβ_). *Q*_intβ_ and *Q*_extβ_ represent a subset of the barrier-free space *Q*_free_. Then, *Q*_intβ_: = {*q* $\in$ *Q*_free_: *H*_q, β_ ⊆ *Q*_free_} and *Q*_extβ_: = *Q*_free_\*Q*_intβ_ [18].

#### Definition 2

(*Strong β-clearance*) For a feasible path *α*: [0,1], only when all points on the path belong to *Q*_intβ,_ that is, *α*(s) $\in$ *Q*_intβ_; ∀s $\in$ [0,1], the path has strong η-clearance.

#### Definition 3

(*Weak β-clearance*) A path *α*_1_: [0,1] has weak β-clearance when there exists a path *α*_2_: [0, 1] and function *φ*: [0, 1] such that *φ* (0) = α_1_, *φ* (1) = *α*_2,_ and for *τ* $\in$ (0, 1], *φ* (*τ*) has strong β-clearance.

#### Definition 4

(*Asymptotic optimality*) When the number of samples is infinite, ALG is asymptotically optimal if it returns a feasible path containing the minimum cost solution. This can be expressed as

$$  {\mathbb{P}}\left( {\mathop {\lim }\limits_{{n \to 8}} \sup Y_{n}^{{{\text{ ALG}}}}  = M*} \right) = 1 $$

where M* represents the optimal path cost. P_RRT* is only an improvement upon the random sampling method of the RRT* algorithm based on the RRT* algorithm, and this operation does not affect the asymptotic optimality of the algorithm. Therefore, P-RRT* inherits the property of RRT* asymptotic optimality [18]. The improved P_RRT* algorithm in this paper is an optimized version of the P-RRT* algorithm, so the improved P_RRT* in this paper also has the property of asymptotic optimality.

### Fast convergence to optimal solution

For Question 3 (mentioned in the definition of the path planning problem), the algorithm is required to find the optimal path in the shortest time *t* $\in$ *T*.

#### Definition 5

(*Optimal path planning*) If a path α* is collision-free and has weak β-clearance, then the path is the optimal path.

#### Theorem 2

(*(Potential guided sampling heuristic RGD(q*)The RGD(*q*) step in P_RRT* guides random samples to the target area for sampling so that $\mathbb{P}(q_{{{\text{prand}}}}  \in Q_{{{\text{ext}}\beta }} )$ > 0.

According to Theorem 2 and Definition 5, P_RRT* has the property of quickly converging to the optimal solution [7, 18]. The improved P_RRT* in this paper further optimizes the search process of P_RRT*. The search time is reduced while remaining unchanged with the rest of the P_RRT* process. Therefore, the improved P_RRT* and P_RRT* have the same property of quickly converging to the optimal path solution.

### Computational complexity

Computational complexity analysis of the improved P_RRT* algorithm is performed. Theorem 3 shows that the improved P_RRT* algorithm has a computational complexity similar to that of P_RRT*.

#### Theorem 3

There is a constant a $\in$ R_+_, such that the following formula remains true:

$$ {\text{lim}}_{n \to \infty } {\mathbb{E}}\left[ {\frac{{S_{n}^{{{\text{Improved P\_RRT*}}}} }}{{S_{n}^{{{\text{P\_RRT*}}}} }}} \right] \le a $$

#### Proof of Theorem 3

Since the improved P_RRT* algorithm only improves the selection method and expansion strategy of the nearest neighbour node of the P_RRT* algorithm, it does not increase the number of samples but optimizes the P_RRT* algorithm, which does not greatly affect the complexity, so the improved P_RRT* algorithm and P_RRT* algorithm have similar computational complexity.

## Deletion of redundant nodes and the maximum curvature constraint

### Redundant node deletion operation

**Fig. 9:**
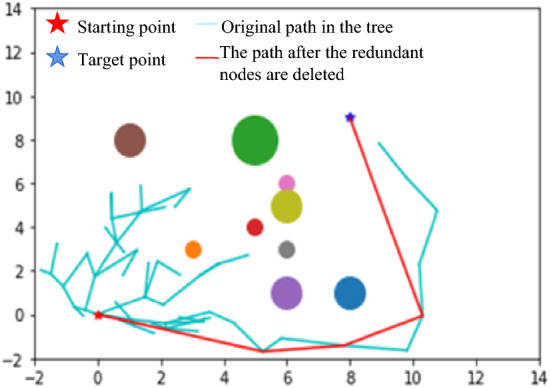
Operation diagram of the path with the redundant nodes deleted

Due to the random expansion of the algorithm, it will inevitably cause the path to become tortuous and include many unnecessary turning points. For a robotic arm, the redundant turning points will cause unnecessary energy loss, cause wear and reduce the service life of the robotic arm. Therefore, this article adopts an operation to remove redundant points [28, 29]. The redundant node removal operation is performed on the generated path. The specific operation starts at the starting point, connects the subsequent way points, abandons the second way point, and connects to the third way point. If the path does not collide with obstacles, the second node is determined to be redundant and is deleted. The starting node is then connected to the fourth node to determine whether there is a collision; if there is no collision, the third node is deleted. The process continues according to this method; if there is a collision, the node is retained and used as the starting point to make the above decisions until the target point is reached. This will generate a path that removes the redundant nodes, as shown in Fig. 9.

### Maximum curvature constraint

**Fig. 10:**
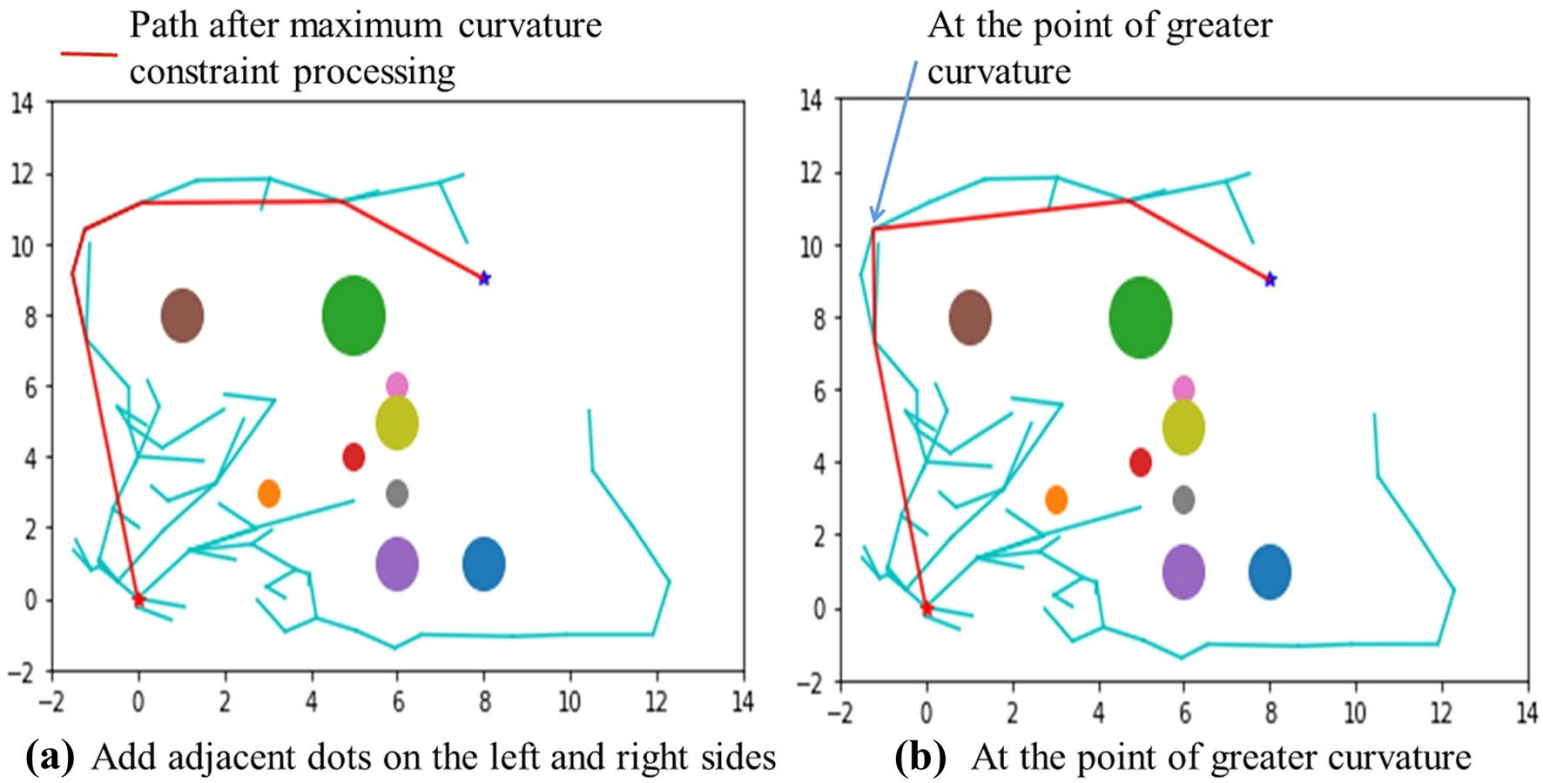
Maximum curvature constraint operation diagram

In the redundant node deletion operation section of this article, a path after removing redundant nodes is obtained, and the path is smoother. The obtained path may have an excessively large rotation angle, which may cause impact damage to the robotic arm during operation, can seriously affect the service life of the robotic arm and is not suitable for an actual application of a robotic arm. Therefore, the maximum curvature constraint method [30, 31] is used to smooth the path. According to the data analysis, the cosine value of the maximum corner angle is 0.707. The cosine value of each angle between the paths is calculated in order from the starting point of the obtained path with the redundant points removed. If the value is greater than 0.707, the child node on the right side and the parent node on the left side of this vertex from the original path are added as standards; that is, the parent nodes on the left side and the child nodes on the right side are added. As shown in Fig. 10b, where the curvature is relatively large, the left and right adjacent points of this point from the original path are added to the left and right sides of the point, as shown in Fig. 10a. This is a preparatory step for the Bezier curve fitting method described below.

### Bezier curve

**Fig. 11:**
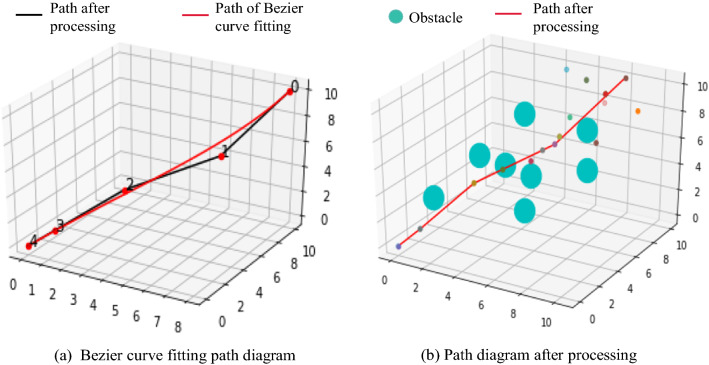
Bezier curve fitting diagram

The path after the deletion of the redundant nodes and the maximum curvature constraint operation is already a relatively smooth path, as shown in Fig. 11b, but in practical applications, it is necessary to minimize the impact damage at the turning point. Therefore, in this section, the Bezier curve method [32, 33] is used for the final smoothing of the path; it makes the path smoother and more suitable for the application of a robotic arm in actual scenes. With a Bezier curve of order *n*, which includes *n* + 1 nodes, the formula *C*(*u*) is as follows:

4 $$ C\left( u \right) = \sum\limits_{{i = 0}}^{n} {B_{{n,i}} } \left( u \right) \times p_{i} ,{\text{ }}u{\mkern 1mu}  \in [0,1] $$

In the formula, *p*_*i*_ represents *n* + 1 points in the space, the weight coefficient B_*n,i*_(*u*) with the parameter *u* represents the Bernstein basis function, and the formula is calculated as follows:

5 $$ B_{{n,i}} \left( u \right) = \frac{{n!}}{{i!\left( {n - i} \right)!}}u^{i} (1 - u)^{{n - i}}  $$

The final generated curve is composed of *n* + 1 nodes, and these nodes are called control points. When *u* = 0 and *u* = 1, they are located at the start and end points, respectively. The obtained path diagram fitted by the Bezier curve is shown in Fig. 11a. It can be determined from the diagram that there is a slight difference between the fitted path and the original path, which may lead to collisions. Under numerous experimental tests, the probability of collision is 0.

## Experiments and analysis

In this section, the improved P_RRT* algorithm is experimentally verified, the improved P_RRT* algorithm is compared with the existing RRT, RRT* and P_RRT* algorithms in the same three-dimensional environment, and the superiority, effectiveness and reliability of the improved algorithm are verified. This experiment is a Python and ROS simulation experiment conducted in the Windows 10 environment of an HP Intel (R) Core (TM) i5-6500 CPU @3.20 GHz and 3.19 GHz with 4.

GB memory in the laboratory. The initial node coordinates are set to [0,0,0], the end node coordinates are [8, 10, 10], the random expansion step length *ρ* is 1.0, *λ* = 0.02, *k* = 80, d*_obs_ = 0.1, the adjacent circle radius r and other parameters are the same, the radius of the adjacent circle used for reselecting the parent node is 2 (*r*_1_ = 2), and the radius of the adjacent circle used for the rewiring operation is 1 (*r*_2_ = 1). Then, an efficient and collision-free path is planned from the start point to the end point. Furthermore, this paper uses the UR5 manipulator of UAO to implement and verify the simulation experiment. When the search time exceeds 100 s, the search is unsuccessful; otherwise, it is successful. This article mainly compares the average search time, average number of sampling nodes, average path length, search success rate and other indicators with those of the RRT, RRT* and P_RRT* algorithms for comparative experimental analyses.

### Python experiment analysis

#### Experiment 1: Algorithm comparison experiment with the same number of obstacles

The superiority of the proposed algorithm will be verified through comparison. Specific experimental contents are as follows: the RRT, RRT*, and P_RRT* algorithms are compared in environments with the same number of randomly distributed obstacles. Each group of experiments is performed 200 times. Due to the randomness of the algorithm, the average value is taken for comparison, as shown in Table 1.

**Table 1:** Comparison of the algorithms under the same number of obstacles

	Average search time	Average number of sampling nodes	Average path length	Search success rate
RRT	80.98	297.30	28.86	70%
RRT*	36.21	187.40	27.28	100%
P_RRT*	17.92	194.81	23.86	100%
Improved P_RRT*	3.01	61.35	21.75	100%

According to the data in Table 1, the average search time and the average number of sampling nodes of the classic RRT algorithm are very large compared to those of the other three algorithms. The average length of the path is also the longest, and the search success rate is 70%. The search time of the RRT* algorithm is the second slowest. Compared with the RRT algorithm, the average search time and the average number of sampling nodes were significantly reduced. The average search time of the P_RRT* algorithm compared to those of the RRT and RRT* algorithms also changed significantly, but the average number of sampling nodes remained high, which inevitably led to the requirement for more memory and consumption of more calculation space by the algorithm. Compared with the other three algorithms, the improved P_RRT* algorithm shows a significant reduction in the average search time and average number of sampling nodes, and its average path length is also the shortest. The superiority of the improved algorithm is, therefore, obvious.

#### Experiment 2: Algorithm comparison experiment with different obstacle numbers

The effectiveness of the proposed algorithm will be compared. Specific experimental contents are as follows: the RRT, RRT*, and P_RRT* algorithms are compared in environments with different numbers of randomly distributed obstacles. Each group of experiments was carried out 200 times, and the average of the results was taken, as shown in Table 2.

**Table 2:** Comparison of the algorithms under different obstacle numbers

Obstacle	Average search time	Average number of sampling nodes	Average path length	Search success rate
	RRT	RRT*	P_RRT*	Improved P_RRT*
6	47.57 168.80 28.40 75%	27.78 147.90 26.50 100%	14.94 177.60 23.70 100%	3.00 52.95 21.15 100%
8	80.98 297.30 28.86 70%	36.20 187.40 27.28 100%	17.92 194.80 23.86 100%	3.01 61.35 21.75 100%
10	106.10 408.50 29.57 65%	40.12 186.00 27.14 100%	19.52 212.20 24.23 100%	3.20 66.10 21.99 100%
12	109.20 427.60 28.28 55%	55.05 187.30 27.76 80%	20.68 234.60 25.36 100%	3.70 72.50 22.75 100%

Table 2 shows that the search time of the classic RRT algorithm is relatively small when there are few obstacles; when there are many obstacles, the average search time and the average number of sampling nodes increase rapidly, and the search success rate is significantly reduced. With the gradual increase in the number of obstacles, the average search time of the RRT* algorithm increases significantly, the search success rate is reduced, and the average number of sampling nodes is increased. Compared with the RRT and RRT* algorithms, the average search time and average path length of the P_RRT* algorithm are significantly improved; however, as the number of obstacles gradually increases, the average number of sampling nodes also greatly increases, the convergence is reduced, and an increasing amount of memory space is consumed. The improved P_RRT* algorithm can still maintain its search efficiency as the number of obstacles gradually increases. The average search time is small and stable. The average number of sampling nodes is also significantly reduced. The algorithm has a faster convergence rate, and its average path length is greatly reduced compared with the RRT, RRT* and P_RRT* algorithms. The effectiveness and excellent results of the improved algorithm are obvious.

#### Experiment 3: Algorithm comparison experiment with two environmental maps

**Fig. 12:**
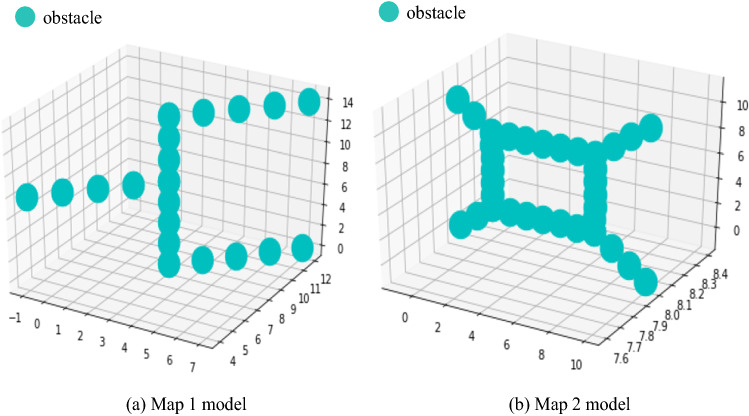
Two different environmental map models

The reliability of the proposed algorithm will be compared with existing algorithms. A comparative experiment with each algorithm is carried out on two environmental maps. The two environmental map models are shown in Fig. 12, and an experimental analysis is performed with the RRT, RRT* and P_RRT* algorithms, as shown below.

#### Map 1

**Fig. 13:**
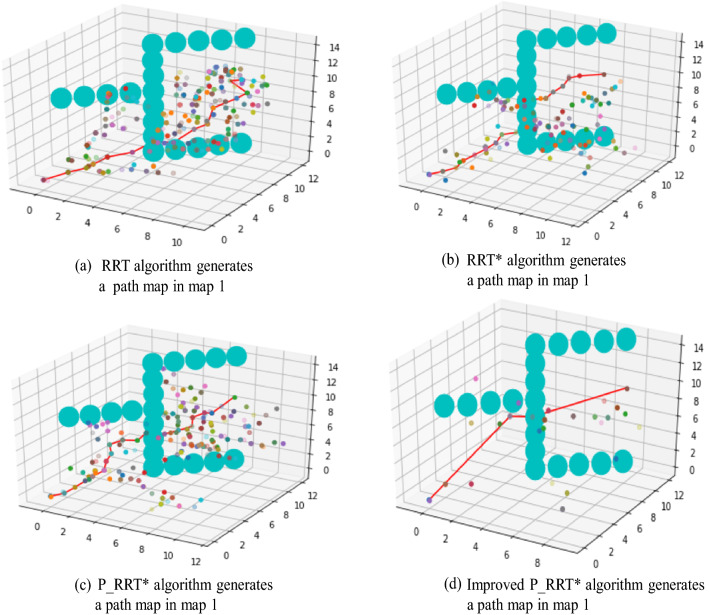
Different algorithms generate path maps in Map 1

Under the condition that the starting point and target point are the same, the results of the RRT, RRT*, and P_RRT* algorithms are compared. Each group of experiments is carried out 200 times, and the average value of the results is taken, as shown in Table 3. The path diagrams generated by different algorithms are shown in Fig. 13a–d.

**Table 3:** Algorithm comparison with respect to Map 1

Algorithm	Average search time	Average number of sampling nodes	Average path length	Search success rate
RRT	76.38	110.23	25.17	70%
RRT*	52.99	62.20	24.49	80%
P_RRT*	29.20	73.20	21.86	100%
Improved P_RRT*	4.51	11.80	17.66	100%

#### Map 2

**Fig. 14:**
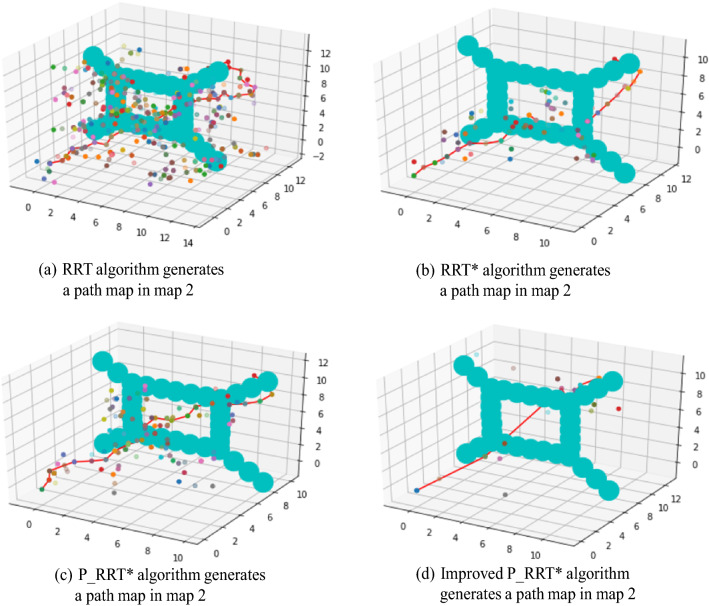
Different algorithms generate path maps in Map 2

Under the condition that the starting point and target point are the same, the results of the RRT, RRT*, and P_RRT* algorithms are compared. Each group of experiments is performed 200 times, and the average value of the results is taken, as shown in Table 4. The path diagrams generated by different algorithms are shown in Fig. 14a–d.

**Table 4:** Algorithm comparison with respect to Map 2

Algorithm	Average search time	Average number of sampling nodes	Average path length	Search success rate
RRT	83.89	139.50	26.49	65%
RRT*	62.72	93.00	25.20	75%
P_RRT*	46.36	108.52	22.80	95%
Improved P_RRT*	8.30	23.90	18.70	100%

According to the data in Tables 3 and 4, when there are many obstacles on Maps 1 and 2 and the environment is complex, the average search time of the RRT algorithm is very large. The required times are 76.38 s and 83.89 s for Maps 1 and 2, respectively. The RRT* algorithm has search times of 52.99 s and 62.72 s, the P_RRT* algorithm has search times of 29.2 s and 46.36 s, and the improved P_RRT* algorithm has minimum search times of 4.51 s and 8.3 s for Maps 1 and 2, respectively. The planning efficiency and search success rate of the RRT algorithm are very low, and the average number of sampling nodes is very high, resulting in substantial memory usage. Compared with the RRT algorithm, the average search time and average sampling node number of the RRT* algorithm are reduced; however, compared with P_RRT* and the improved P_RRT* algorithm, they are still very high, and the search success rate is also low. The P_RRT* algorithm has improved average search times and average path lengths compared to those of the RRT and RRT* algorithms, but the average number of sampling nodes remains very large. Compared with the improved P_RRT* algorithm proposed in this paper, the P_RRT* algorithm still exhibits the shortcomings of low efficiency, a large memory footprint, and long tortuous paths. In this paper, the improved P_RRT* algorithm has a smaller average search time and average number of sampling nodes. It shows a more prominent effect in a complex multiobstacle environment, and its search path is shorter and smoother. The reliability of the improved algorithm is obvious through the comparative analysis of two different environment map experiments.

### ROS simulation experiment analysis

**Fig. 15:**
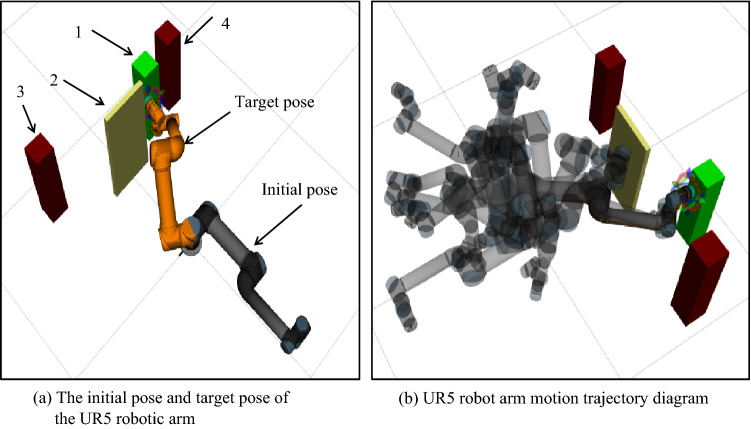
Motion simulation diagram of the UR5 robotic arm

**Fig. 16:**
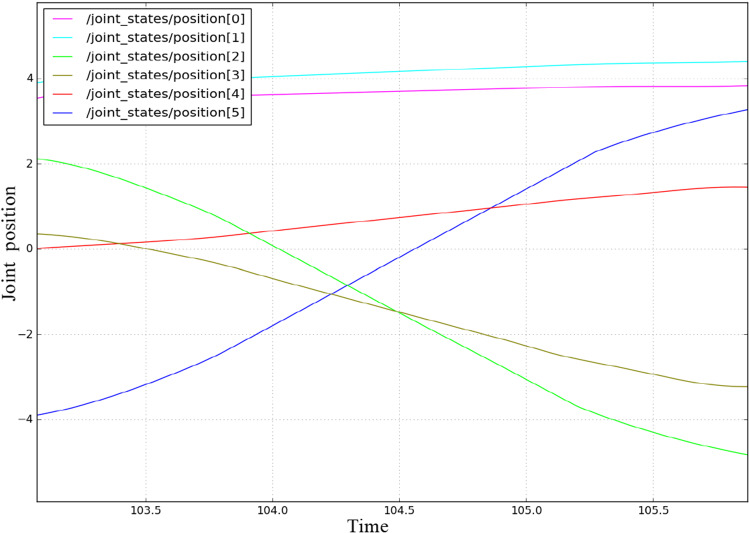
Change in the joint position of the robotic arm

To combine theory with reality and to verify the feasibility of the algorithm in this article, this section uses the UR5 manipulator of UAO to perform a simulation analysis in the ROS. The UR5 manipulator is a six-degree-of-freedom articulated manipulator. First, the environment scene is built in MoveIt, the rviz tool in ROS is used to visualize the demonstration, the robotic arm model is loaded, and the error transformation matrix is used to compensate for the docking error of the mobile robotic arm. The obstacles, starting point pose, and target point pose are set. Here, the starting point and target point pose are the poses after error compensation. The improved P_RRT* algorithm is added to the Open Motion Planning Library (OMPL), and the corresponding ymal file is modified. The Kinematics and Dynamics Library (KDL) solver in MoveIt is used to solve each joint angle, and finally, a collision-free path that meets the requirements is obtained, as shown in Fig. 15b. In Fig. 15a, 1 represents the target and 2, 3, and 4 represent the obstacles near the target. The horizontal manipulator represents the initial pose and reaches the target pose that coincides with the yellow pose. The position values of each joint at the start and end points of the robotic arm are shown in Table 5. The trajectory of the robotic arm is shown in Fig. 15b. Figure 16 shows the position change diagram of each joint during the simulation of the manipulator. The figure shows that the manipulator runs smoothly in the simulation experiment of the improved P_RRT* algorithm, and the algorithm meets the movement needs of a real manipulator. The simulation experiment verifies the feasibility of the algorithm in this paper.

**Table 5:** Position values of each joint at the start and target points of the robotic arm

	Joint 0	Joint 1	Joint 2	Joint 3	Joint 4	Joint 5
The starting position	0.00112	0.00365	1.979e−05	0.000136	− 1.0392e−05	9.0132e−06
The target position	2.40849	6.10617	− 6.17202	3.42690	− 0.86144	− 0.57220

To verify the superiority of the improved algorithm, the proposed algorithm was compared with the RRT, RRT* and P_RRT* algorithms. Under the same obstacle conditions mentioned above, 20 simulation experiments were performed for each algorithm, and the average of the results was used, as shown in Table 6. According to the table, the improved P_RRT* algorithm still has high motion planning efficiency in the simulation experiment. The superiority of the improved algorithm is obvious.

**Table 6:** Comparison of the algorithms in the same ROS obstacle environment

	Average search time	Search success rate
RRT	11.26	100%
RRT*	10.65	100%
P_RRT*	8.36	100%
Improved P_RRT*	4.83	100%

## Conclusion

The improved P_RRT* algorithm proposed in this paper improves the P_RRT* algorithm’s shortcomings of low search efficiency and slow convergence speed. The superiority, effectiveness, reliability and feasibility of the improved algorithm in this paper are verified through experimental comparisons and analyses. The steps of the improved algorithm are as follows:

1. The same random sampling strategy as P_RRT* is used;
2. The selection strategy of the nearest neighbour node is introduced. With a certain probability, the traditional Euclidean distance method and the minimum cost function *C* (*q*) are used alternately to select the nearest neighbour node so that the algorithm can avoid obstacles more effectively;
3. The two expansion strategies for new nodes are introduced, and on the basis of the traditional new nodes adopting a target-biased strategy expansion, the second expansion of new nodes is performed, which more effectively improves the efficiency of the algorithm;
4. The reselection of the parent node from the RRT* algorithm and the rerouting operation from the RRT* algorithm are performed on the new node, and an optimal path is obtained.
5. The redundant node removal operation and the maximum curvature constraint operation are performed on the path after reselecting the parent node and the rerouting operation. The redundant nodes in the path are removed, and the maximum curvature constraint makes the path smoother by adding points from the original path to the left and right sides of the turning point at a corner with a larger curvature.
6. After the removal of redundant points and the maximum curvature constraint operation, the path is fitted with the Bezier curve, and a smooth path that is more consistent with the manipulator motion is obtained.

Through Python experiments, it can be determined that the improved algorithm has higher search efficiency and higher convergence rates, the search time and path length are effectively reduced, and the average number of sampling nodes is significantly reduced, which reduces the computer's memory consumption. The comparison of the average search times, the average search success rates, and the motion trajectories of the robotic arm obtained by the other algorithms in the ROS simulation experiment also proves the superiority of the improved algorithm.

This paper studies the spatial path planning of a robotic arm; the method can be applied in unstructured environments, such as logistics storage, material stacking, and cargo handling. In the future, the dynamic path planning of a robotic arm will be studied in a dynamic environment.

## Data Availability

All data generated or analysed during this study are included in this published article [and its supplementary information files].

## Code availability

All data, models, and code generated or used during the study appear in the submitted article.

## References

[1] Suarez A, Heredia G, Ollero A (2018). Physical-virtual impedance control in ultralightweight and compliant dual-arm aerial manipulators. IEEE Robot Autom Lett.

[2] Zeng A, Yu K T, Song S (2017) Multi-view self-supervised deep learning for 6D pose estimation in the Amazon Picking Challenge[C]. In: 2017 IEEE Int Conf robot Autom (ICRA) IEEE 1386–1383

[3] Chen F, Selvaggio M, Caldwell DG (2018). Dexterous grasping by manipulability selection for mobile manipulator with visual guidance. IEEE Trans Ind Inf.

[4] Wang M, Hou Z (2018). Continuous trajectory point control research of six degree of freedom mechanical intelligent arm position. Int J Precis Eng Manuf.

[5] Lavalle S M (1998) Rapidly-exploring random trees: a new tool for path planning. 1998

[6] Li Y Z, Wang S T, Jiang L Q, Meng J, Xie Y L (2021) Mobile manipulator motion planning based on sparse node RRT. China Mech Eng 1–8

[7] Li Y, Wei W, Gao Y (2020). PQ-RRT*: an improved path planning algorithm for mobile robots. Expert Syst Appl.

[8] Wang W, Zuo L, Xu X (2018). A learning-based multi-RRT approach for robot path planning in narrow passages. J Intell Robot Syst.

[9] Jeong IB, Lee SJ, Kim JH (2019). Quick-RRT*: triangular inequality-based implementation of RRT* with improved initial solution and convergence rate. Expert Syst Appl.

[10] Pérez-Higueras N, Caballero F, Merino L (2018). Teaching robot navigation behaviors to optimal RRT planners. Int J Soc Robot.

[11] Li Y, Cui R, Li Z (2018). Neural network approximation based near-optimal motion planning with kinodynamic constraints using RRT. IEEE Trans Ind Electron.

[12] Chen L, Shan Y, Tian W (2018). A fast and efficient double-tree RRT*-like sampling-based planner applying on mobile robotic systems. IEEE/ASME Trans Mechatron.

[13] Hidalgo-Paniagua A, Bandera JP, Ruiz-de-Quintanilla M (2018). Quad-RRT: a real-time GPU-based global path planner in large-scale real environments. Expert Syst Appl.

[14] Chao N, Liu Y, Xia H (2018). Grid-based RRT* for minimum dose walking path-planning in complex radioactive environments. Ann Nucl Energy.

[15] Ryu H, Park Y (2019). Improved informed RRT* using gridmap skeletonization for mobile robot path planning. Int J Precis Eng Manuf.

[16] Kiani F, Seyyedabbasi A, Aliyev R (2021). Adapted-RRT: novel hybrid method to solve three-dimensional path planning problem using sampling and metaheuristic-based algorithms. Neural Comput Appl.

[17] Qi J, Yang H, Sun H (2020). MOD-RRT*: a sampling-based algorithm for robot path planning in dynamic environment. IEEE Trans Ind Electron.

[18] Qureshi AH, Ayaz Y (2016). Potential functions based sampling heuristic for optimal path planning. Auton Robot.

[19] Hu Y, Su H, Fu J (2020). Nonlinear model predictive control for mobile medical robot using neural optimization. IEEE Trans Ind Electr.

[20] Hu Y, Li J, Chen Y (2021). Design and control of a highly redundant rigid-flexible coupling robot to assist the COVID-19 oropharyngeal-swab sampling. IEEE Robot Automat Lett.

[21] Jordan M, Perez A (2013) Optimal bidirectional rapidly-exploring random trees.

[22] Wei K, Ren B (2018). A method on dynamic path planning for robotic manipulator autonomous obstacle avoidance based on an improved RRT algorithm. Sensors.

[23] Nasir J, Islam F, Malik U (2013). RRT*-SMART: a rapid convergence implementation of RRT. Int J Adv Rob Syst.

[24] Tan JH, Pan B (2020). Robot path planning based on improved RRT * FN algorithm. Control decis.

[25] Karaman S, Frazzoli E (2011). Sampling-based algorithms for optimal motion planning. Int J Robot Res.

[26] Yuan C, Zhang W, Liu G (2019). A heuristic rapidly-exploring random trees method for manipulator motion planning. IEEE Access.

[27] Zhang WM, Fu SX (2021). Path planning of mobile robot based on improved RRT* algorithm. J Huazhong Univ Sci Technol.

[28] Shi Y, Li Q, Bu S (2020) Research on intelligent vehicle path planning based on rapidly-exploring random tree. Math Probl Eng 1–14

[29] Yang SM, Lin YA (2021). Development of an improved rapidly exploring random trees algorithm for static obstacle avoidance in autonomous vehicles. Sensor.

[30] Gim S, Adouane L, Lee S (2017). Clothoids composition method for smooth path generation of car-like vehicle navigation. J Intell Robot Syst.

[31] Ding Y, Xin B, Chen J (2019). Curvature-constrained path elongation with expected length for Dubins vehicle. Autom.

[32] Xiong X, Min H, Yu Y (2021). Application improvement of A* algorithm in intelligent vehicle trajectory planning. Math Biosci Eng MBE.

[33] Lee H, Kim H, Kim HJ (2016). Planning and control for collision-free cooperative aerial transportation. IEEE Trans Autom Sci Eng.

